# Emergency care in India beyond 75 years of independence – problems and solutions

**DOI:** 10.7189/jogh.13.03015

**Published:** 2023-04-14

**Authors:** Asit Misra, Dolly C Yadav, Tamorish Kole

**Affiliations:** 1 Department of Surgery (Division of Emergency Medicine) and Gordon Center for Simulation and Innovation in Medical Education, University of Miami Miller School of Medicine, Miami, Florida, USA; 2 Emergency Department, Queen’s Medical Centre, Nottingham University Hospitals NHS Trust, Nottingham, UK; 3 Medhavi Skillversity PLC, Gurgaon, India; **Correspondence to:** Asit Misra Department of Surgery (Division of Emergency Medicine) and Gordon Center for Simulation and Innovation in Medical Education, University of Miami Miller School of Medicine SCRC, 1st Floor 1120 NW 14th Street Miami, Florida USA asit.misra@med.miami.edu

**Figure:**
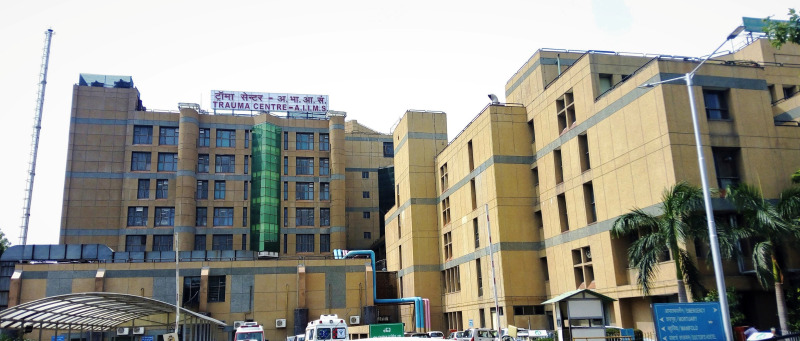
Photo: JPNA Trauma Center by M. Vishnoi, used under the Creative Commons Attribution-Share Alike 4.0 International license, cropped from original.

On August 15, 2022, India celebrated 75 years of independence. Over the past 75 years, India has focussed on its scientific progress, including health care, leading to Ayushman Bharat Pradhan Mantri Jan Aarogya Yojana (PMJAY), the world’s most ambitious national program for universal health coverage [1]. This initiative aligns with the Sustainable Development Goals (SDGs) that aim to achieve universal health coverage (UHC) globally by 2030. In other words, delivering high-quality health services to everyone such that people across the globe receive promotive, preventive, curative, rehabilitative, or palliative care, as required, without experiencing monetary adversities [2]. Vital Universal Health Care stands on three pillars: primary care, emergency care (EC), and definitive care, including secondary and tertiary level care (**Figure 1**).

**Figure 1:**
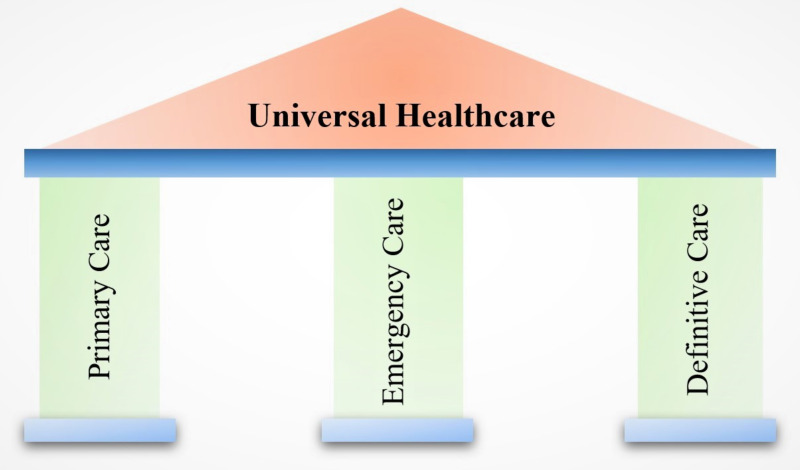
The three pillars of universal health care.

## EMERGENCY MEDICINE – THE PATH TRAVELLED SO FAR

As recently as the 1950s, the “emergency room” was often just a hospital room reserved for emergency cases. The room was staffed by either inexperienced interns or rotating members of the hospital’s medical staff – regardless of their training, expertise, or interest. Rising demand in the years following World War II quickly outstripped the capacity of this haphazard approach to emergency staffing. The United States of America was one of the early adopters of emergency medicine (EM) as a speciality. The American College of Emergency Physicians was formed in 1968. In 1970 the first formal EM residency program was started at the University of Cincinnati in the United States [3].

In India, in 1982, Gautam Sen, a Professor Emeritus of Surgery at Grant Medical College University of Mumbai, founded the Association for Trauma Care of India (ATCI). This is the first organisation to launch an emergency medical service (EMS) under the “Golden Hour Project”. Later in 2000, the Society for Emergency Medicine, India, was formed, which paved the way for modern Emergency Care (EC) in India. The specialty of EM was officially recognised on July 21, 2009, as India’s 30th discipline for postgraduate medical education. Finally, in 2019, the government of India made it mandatory for all medical colleges to have an EM department by 2022 to improve EC across the country [4].

## NEED FOR EMERGENCY CARE

In 2005, the World Health Organization (WHO) published recommendations for actions regarding emergency medical systems in low- and middle-income countries (LMICs). It was observed that emergencies are inevitable occurrences, and they utilised assets irrespective of the system’s ability to attain favourable outcomes. However, improved planning, training, and capacity building resulted in the development of evidence-based EC systems tailored to the needs of LMICs. This approach boosts health equity as well as cost-effectiveness. Thus, building organised EC is an essential contributor to decreasing avoidable deaths and disabilities in LMICs [5].

In LMICs, it is gauged that by improving prehospital and EC, deaths may be reduced by half. This is especially true in cases of injuries, infections, exacerbations of non-communicable diseases, and complications of pregnancy. Considering the above, at the 72nd World Health Assembly on May 30, 2019, it was pledged to assist countries in establishing timely care for the acutely ill and injured [6]. In fact, WHO Director-General Dr Tedros Adhanom Ghebreyesus emphasised that “no one should die for the lack of access to emergency care, an essential part of universal health coverage. We have simple, affordable, and proven interventions that save lives. People worldwide should have access to the timely, lifesaving care they deserve” [6]. The fulfilment of WHO’s 13^th^ General Programme of Work 2019-2023 is made feasible by improving the essential health services coverage and integrating the delivery of services because a well-organised EC system includes universal health coverage. Additionally, a well-established EC system augments the attainment of several other SDG targets, namely, road safety, maternal and child health, non-communicable diseases, infectious diseases, disaster and violence [6].

India is a signatory to this resolution and is committed to achieving this target. However, a population of 1.38 billion, a high trauma mortality rate, an increasing burden of emergencies, and frequent natural disasters pose a challenge to building an emergency care system for all citizens in the country. According to the World Bank, India is home to just 10% of the world’s registered vehicles, accounting for 22% of traffic deaths. Notably, despite COVID-19 restrictions, more than 131 000 people lost their lives in road accidents in 2020. Furthermore, road accident deaths have increased by 7% in the first five months of 2022, indicating an immediate need to strengthen post-crash (medical) care [7].

## CURRENT STATUS OF EMERGENCY CARE IN INDIA

On December 10, 2021, NITI Aayog, the apex public policy think-tank of the Government of India, released two comprehensive reports on the status of a country-level secondary and tertiary level care, and district-level emergency and injury care in India. These assessments highlighted the disparity in delivering quintessential care due to shortcomings in prehospital EM (ambulance) facilities, health framework, workforce, and medical supplies [8].

As per these reports, most hospitals lacked the presence of general doctors (practitioners), specialists, and nursing staff dedicated to emergency departments (EDs) vis-a-vis the average footfall of patients, even though the hospitals had sufficient overall numbers of required human resources (**Table 1**).

**Table 1:** Summary of the NITI Aayog report on the current status of country-level secondary and tertiary level, and district-level emergency care in India

Focus area	District hospitals (government facilities)	Secondary and tertiary level hospitals (government facilities)	Secondary and tertiary level hospitals (private facilities)
Percentage of emergency and injury cases	16% of all patients presenting to a health facility and 19%-36% of admissions in district hospitals annually.	9%-13% of all patients presenting to a health facility and 19%-24% of admissions in government hospitals annually.	9%-13% of all patients presenting to a health facility and 31%-39% of admissions in private hospitals annually.
Emergency medical services (ambulance)	88% of hospitals had in-house ambulances; in only 3% of hospitals, trained paramedics were available to assist with ambulance services.	91% of hospitals had in-house ambulances, and only in 34% of hospitals trained paramedics were available to assist with ambulance services.	Provision of specialised care during ambulance transport was largely poor: 19% of hospitals had a mobile Stroke / STEMI (for heart attack) program, with only 4% having a mobile Stroke unit.
Emergency department beds	Only 3%-5% of total hospital beds are available in emergency departments despite the high patient load	Same as district hospitals.	Same as district hospitals.
Emergency care workforce	Most hospitals lacked the presence of general doctors, specialists, and nursing staff dedicated to emergency departments even though the hospitals had a sufficient workforce.	Same as district hospitals.	Same as district hospitals.
Equipment for emergency care	Compliance deficiencies were found with the availability of recommended biomedical and critical equipment (45%-60%).	Satisfactory compliance with the availability of recommended biomedical and critical equipment (68%), with deficiencies found in smaller government hospitals (45%-60%).	Satisfactory compliance with the availability of recommended biomedical and critical equipment (86%-93%).
Financial support for emergency care	None had funds dedicated to emergency care services. A few hospitals received funds as part of delivering trauma care.	Same as district hospitals. The Eastern Zone was the worst affected regarding receiving funds from the central / state government.	Same as government facilities.

In some areas, private hospitals were better than government hospitals. For example, private hospitals fared better than government hospitals in terms of having emergency operative services, mock drills, training programs, regular audits, and referral policies. Private hospitals also ensure effective communication skills amongst caregivers and timely care delivery, translating into higher patient satisfaction levels.

Hospitals conducting structured academic programs in the subject of EM have comprehensive, robust systems in place for efficient patient care services, including critical care and definitive care, tackling imminent disasters, and continuous quality improvement. These systems also ensured effective communication skills amongst caregivers and timely care delivery, translating into higher patient satisfaction levels.

Although the NITI Aayog’s report portrayed that there are certain aspects where the private health system scored better, still public health care is the foundation of the Indian health care system. Along with introducing the National Health Insurance (PM-JAY) policy, the reimbursement mechanism is uniform across the sectors. Therefore, both sectors have started working synergistically to provide better emergency care. Also, these findings are a reminder of decades of challenges and opportunities fundamental to the Indian constitutional right to LIFE (article 21) [9].

## IMMEDIATE NEEDS

**Figure 2:**
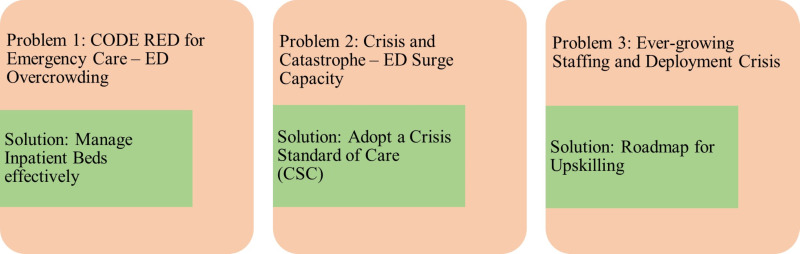
Top three problem areas in the context of emergency care (EC) and suggested solutions.

We identified top three problem areas needing immediate attention in context with EDs and EC in India. Possible solutions are shown in **Figure 2** and discussed below:

### Problem 1: CODE RED for emergency care – emergency department overcrowding

Globally, EDs were initially designed to provide immediate care for patients with time-sensitive conditions. However, it is often seen that EDs receives patients that do not get cared for at other places, in addition to when health systems cannot accommodate these patients elsewhere [10]. This stresses EDs primarily because they are not structured to operate for the services mentioned earlier and mainly because to perform at surge capacity for natural disasters, public health crises, and pandemics, ED expands its functioning with a limited workforce and resources. This is “CODE RED” for emergency care [10]. India has the same universal emergency care problem of ED overcrowding. In a study of a tertiary care centre in India, the overcrowding scales were compared and a similar pattern was seen as in western countries [11]. Another study cited a lack of necessary infrastructure to handle overcrowding [12].

### Solution: Manage inpatient beds effectively

Emergency department overcrowding is an indicator of a flawed or maladjusted hospital and / or health system and hence, requires hospital and / or system response to address it. It is particularly dependent on the availability of adequate inpatient beds. This, notably, is the efficient management of bed stocks with appropriate use, good flow practices, competing uses for beds (e.g. acutely sick vs elective admissions), using step-down units, and proper community care, and not just the accessibility to the physical number of beds [13]. In a nutshell, ED overcrowding is a hospital and / or health system issue, not just an ED issue. Thus, leadership must ensure effective inpatient bed management, more so with the help of newer technologies [14]. The causes and solutions of ED overcrowding lie outside of it [14]. Regarding effective and efficient inpatient bed management, solutions may be aimed at three areas of patient flow – prehospital, in-hospital, and post-hospital. For example, as a part of India’s National Rural Health Mission (NRHM), an accredited social health activist (ASHA worker) is employed in every village in India. ASHA worker focuses on maintaining demographic records, community sensitisation and counselling regarding sanitation, immunisations, family planning, breastfeeding, provision of drugs, and health surveillance of pregnant women and children [15]. They have efficiently monitored the health and growth status of children, reducing the adversities and admissions related to malnutrition by using an app called “Poshan Tracker” [16].

There is an immense scope for improvement in the prehospital ambulance service and emergency care, including the development of a centralised ambulance and referral system along with prehospital staff capacity building that can transport the patient to the nearest and the best-suited hospital mapped on the national network to avoid overcrowding of EDs. The government of India has taken its first step by launching 112 as a single emergency number to avail the benefits of all existing helpline numbers for police, fire, and women helplines in the state [17].

During the COVID-19 pandemic, regional command centres were established that helped monitor the surge and effectively utilised limited inpatient beds. Integrating such a dynamic system with Artificial Intelligence (AI) and machine learning may build a national system equipped for real-time inpatient management by connecting with the centralised emergency response system. Furthermore, delivering high-quality preventive, rehabilitative, and community care across the country would reduce the rates of ED visits unrelated to health emergencies and shorten the length of hospital stay by transferring patient care closer to home for rehabilitation and social support. Community care’s positive effects on healing and reducing morbidity and mortality are well established. Thus, the government of India introduced the Ayurveda, Yoga, Naturopathy, Unani, Siddha, and Homeopathy (AYUSH) bridge course. It aims to train the AYUSH health care professionals to practice modern medicine at mid-levels in the primary, community, and district care centres, thereby opening avenues for the efficient patient care within the community [18].

All the above initiatives have shown that using technology can help improve community health and reduce avoidable hospital admissions to reduce ED overcrowding.

### Problem 2: Crisis and catastrophe – emergency department surge capacity

India has also witnessed catastrophic disasters – earthquakes, tsunamis, cyclones, urban floods, and hospital fires; each resulted in the hampering of the health care framework and a paramount burden on the medical care system. Furthermore, managing these unforeseen calamities has taught us that disasters overburden health systems and adversely affect the assignment of limited supplies and services. However, this is not isolated to natural calamities and is vital during terrorist incidents, especially in the case of the discharge of bioweapon or nuclear devices or slow onset events such as the COVID-19 pandemic. Emergency department is particularly vulnerable to overcrowding in disasters or even in day-to-day situations because of India’s demand-supply mismatch of health care resources [19].

### Solution: Adopt a Crisis Standard of Care

Crisis Standard of Care (CSC) is defined “as a substantial change in usual healthcare operations and the level of care, which is made necessary by a pervasive (e.g., pandemic like COVID-19) or catastrophic (e.g. earthquake, hurricane) disaster.” The continuum of care between routine situations and disasters must be understood and accepted by all stakeholders related to EC [19,20]. Hospitals must prepare for major disruptions in care because of a mass casualty incident by regularly training staff in hospital disaster management and adopting the incident response system of India’s national disaster management authority [21]. Additionally, private and public hospitals should regularly participate in local (citywide) disaster response exercises to evaluate their preparedness.

### Problem 3: Ever-growing staffing and deployment crisis

Due to increasing demand, massive expansion of emergency services, and glaring skill gaps, there is a staff shortage inside and outside the hospital to provide adequate care. This situation is worsened by violence against ED staff, paramedics, and stress due to systemic burnout. According to a survey study, verbal abuse at the workplace was personally experienced by 70% of respondents. India has over 22 official languages and 122 types of spoken languages. Currently, medicine is taught in English in India, and most clinicians are fluent in it, but a large patient population is not. Clinicians being not fluent in the local spoken language can cause miscommunication that may add to verbal abuse and violence against clinicians in the ED [22]. Moreover, recent human resource budget cuts among hospitals toward emergency medicine have resulted in the deployment of junior-level staff in place of seniors, widening skill gaps, increasing the level of stress, and more attrition.

### Solution: Roadmap for upskilling

First, we must address the skill gap; there is no quick fix. While educational pathways for emergency medicine are established now, it is evident that it will take years to match the demand. In the interim, we must establish capacity-building measures in Phygital mode. This was tested and proven very useful during the COVID-19 pandemic.

Second, India’s prevailing scarcity of nursing professionals leads to a poor nurse-to-patient ratio. To address the above and achieve the advisable WHO norms, India needs to add 4.3 million nurses by 2024. Additionally, limited resources are currently available in India for nurses’ training to provide emergency and critical care. This has posed challenges during the COVID-19 pandemic; therefore, there is a dire need to enhance nursing training programs in emergency care [23]. Another area to focus on is communication training and using professional interpreters where language may be a barrier.

Finally, the prehospital care professionals are coming under the ambit of the proposed Allied Health Council. A National Blueprint can only ensure standardisation of care and monitoring of good medical practice for emergency medical care.

## FUTURE DIRECTIONS

So, what can India expect in the next 25 years? Medicine itself will also take a quantum leap with genomics, precision medicine, and robotics. Therefore, EC needs to be redefined by introducing drones, 5G, AI, and blockchain in health care.

### Internet of Things

The connection of devices and services using the internet, or the internet of things (IoT), promises upcoming technological advancement in health care – the internet of health (IoH). The utilisation of IoH automation is more than likely to enhance patient care across the patient journey from prehospital to hospitalisation. Organising real-time consults between in-hospital specialists and prehospital providers in acute stroke is an example of IoT impacting prehospital care [24].

A solution like “FirstNet,” which is the first nationwide network explicitly engineered for first responders in the US, should be developed and implemented in India for paramedics, emergency medical technicians, police officers, firefighters, and emergency medical professionals to access information and to communicate with other first responders.

### Artificial Intelligence

Newer technologies, such as AI and machine learning, are increasingly utilised in medicine. Moreover, emergency medicine is also taking advantage of this automation. The AI solutions available for emergency care can be grouped into three domains: AI in predictive modelling, AI in patient monitoring, and AI in ED operations. In the next two-three decades, we might use AI for large-scale emergency operations across a state, within states, or even nationally [25].

### Drones

Lately, drones are being successfully utilised during medical emergencies. These are being witnessed assisting rescuers, swiftly transporting blood to faraway locations, locating and connecting to casualties in dangerous circumstances or remote locations, and likely triaging patients before they arrive at health care setups, thereby enhancing the time and quality of medical care delivery. For the reasons above, drones are emerging as a complementary component of traditional medical EC. For example, in Sweden, automated external defibrillator was transported by a drone to a casualty in cardiac arrest, and their life was saved [26].

### Augmented Reality-Virtual Reality-eXtended Reality

eXtended Reality (XR) describes a continuum of immersive computing experiences that includes both Augmented Reality (AR) and Virtual Reality (VR). Furthermore, their development in recent years has enabled the implementation of simulation-based education (SBE) beyond the boundaries of the simulation laboratory, allowing SBE to become asynchronously accessible to learners across diverse global locations. Several studies have elicited the role of VR in cardiopulmonary resuscitation (CPR) and advanced cardiac life support training by using avatar-based gamification and interprofessional team training approaches. These SBE approaches successfully inoculate stress among resuscitation team leaders in a psychologically safe learning environment, improve their situational awareness, decrease anxiety, and promote team building [27]. Emergency medicine based solutions are expected in the near future [28].

## CONCLUDING THOUGHTS

One of the many challenges of delivering emergency care has always been managing the unpredictability of patient volume, presentation, and acuity. As we build our next level of emergency care in the next 25 years, we must address more significant health care issues like universal health coverage, technology adoption for faster prehospital care, and a widening health workforce gap. One of the five themes to commemorate India’s 75 years of independence (Azadi Ka Amrit Mahotsav) is “Resolves @ 75”. The theme aims to strengthen commitments to targets and goals encompassing our shared resolve and commitment to influencing the nation’s progress. While there are many facets to this, effective EC for every citizen in India must be included here. EC is considered a safety net for patients. Will the health ecosystem in India; act as a safety net for EC? We believe, yes!
